# Thrips Spatio-Temporal Distribution in Cowpea (*Vigna unguiculata* (L.) Walp.) Flowers Based on the Flower Structures and Floral Development Stage

**DOI:** 10.3390/plants14243753

**Published:** 2025-12-10

**Authors:** Xiaoyun Ren, Yuyin He, Xinbao Wei, Li Zheng, Haitao Yu, Xunbing Huang, Shengyong Wu

**Affiliations:** 1 College of Agriculture and Forestry Science, Linyi University, Linyi 276000, China; renxiaoyunyouxiang@163.com (X.R.); 17861292479@163.com (Y.H.); zhengli64@126.com (L.Z.); 2 State Key Laboratory for Biology of Plant Diseases and Insect Pests, Institute of Plant Protection, Chinese Academy of Agricultural Sciences, Beijing 100193, China; 3 Gansu Fengmiao Ecological Agriculture Development Co., Ltd., Lanzhou 730070, China; weixinbao_bx@163.com; 4 Institute of Plant Protection, Gansu Academy of Agricultural Sciences, Lanzhou 730070, China; yuhaitao1202@126.com; 5 National Hainan Research Institute (Sanya), Chinese Academy of Agricultural Sciences, Sanya 572024, China

**Keywords:** thrips, cryptic habitat, floral development stage, behavioral preference, differential volatiles

## Abstract

*Megalurothrips usitatus* (Bagrall 1913) (MTU) is a major pest of cowpea (*Vigna unguiculata* (L.) Walp.) and shows a strong preference for floral tissues. To clarify the spatiotemporal distribution of thrips, we conducted a detailed survey of their abundance in flowers of different developmental stages—sampled in the morning (preanthesis flowers, PAF; open flowers, OPF; postanthesis flowers, PoAF) and at dusk (preanthesis flowers scheduled to open the next morning, PAF-D; closed flowers, CF). Behavioral responses of MTU to floral volatiles from these stages were evaluated using a Y-tube olfactometer, followed by chemical analysis via gas chromatography–mass spectrometry (GC-MS). The results indicate that 58.3% of adults sheltered in keel petals, while 76.7% of nymphs aggregated inside the diadelphous stamens. Thrips abundance on OPF in the morning was significantly higher than on PAF or PoAF, but did not differ significantly from that on CF. Olfactometric assays demonstrated a clear preference of MTU for OPF, which emitted a greater number and higher concentrations of volatile compounds compared to PAF, PAF-D, CF, and PoAF. Together, these findings reveal distinct spatiotemporal dynamics of thrips in relation to cowpea flower development, underscoring the role of floral age in driving host-switching behavior.

## 1. Introduction

Thrips are economically important pests worldwide and can cause vast losses to crops by both direct and indirect damage [1,2]. Direct damage is caused through feeding and oviposition: nymphs and adults pierce and suck fluids from leaves, flowers, growing tips, and fruits, while females deposit eggs into plant tissues [3,4]. Thrips can also transmit plant viruses, such as tomato spotted wilt orthotospovirus (TSWV), tomato yellow ring virus (TYRV), and tobacco streak virus (TSV), causing damage to plants indirectly and reducing both crop quality and yields [4,5,6]. The small-sized body, cryptic damage habits, and high reproductive rate of thrips contribute to their persistent status as a major pest [2,3,4,7], and pesticide-dependent management is frequently applied, leading to thrips developing resistance to pesticides [3,8]. Terminal and unopened flower buds can be utilized by thrips as habitats [9], and thrips also show a preference for residing in the crevices of artificial flowers [10]. These behaviors indicate that thrips exploit structural traits of plants, which complicates detection and often results in monitoring failures and untimely control.

Thrips abundance is highly correlated with plant phenology [11,12,13,14] and prefers aggregating in flowers [15,16]. Flowers offer nutritional benefits, providing sugars and amino acids present in pollen and nectar, which support population growth in thrips [17]. Previous studies have shown that the floral traits (flower corolla, size, color, floral volatiles, etc.) [18,19,20,21,22,23,24,25,26] play a significant role in thrips’ foraging for energy-rich diets. Moreover, differential volatile profiles from flowers or different floral stages have been shown to mediate insect behavior [27]. For example, population levels of *Thrips tabaci* (Lindeman 1889), *Frankliniella occidentalis* (Pergande 1895) (western flower thrips, WFT), and *Frankliniella schultzei* (Trybom 1910) correlate with flowering periods of plants [13]. Both WFT and *T. tabaci* show a high preference to flowering plants that emit high levels of fragrant volatiles [14], and 2,4-decadienal identified from flowering *Baccaurea lamiflora* (Lour. 1790) has been demonstrated to attract *Megalurothrips usitatus* (Bagrall 1913) (MTU) in field conditions [27]. Additionally, floral structures can influence the within-flower distribution of thrips [9,28]. For instance, in open flowers, *F. schultzei* tends to aggregate more frequently in the mid and apical sections [28]. Despite the broad host range of thrips, such as MTU and WFT [4,29], which are associated with diverse floral morphologies [18,30], their distribution patterns across specific floral structures remain underexplored.

**Figure 1:**
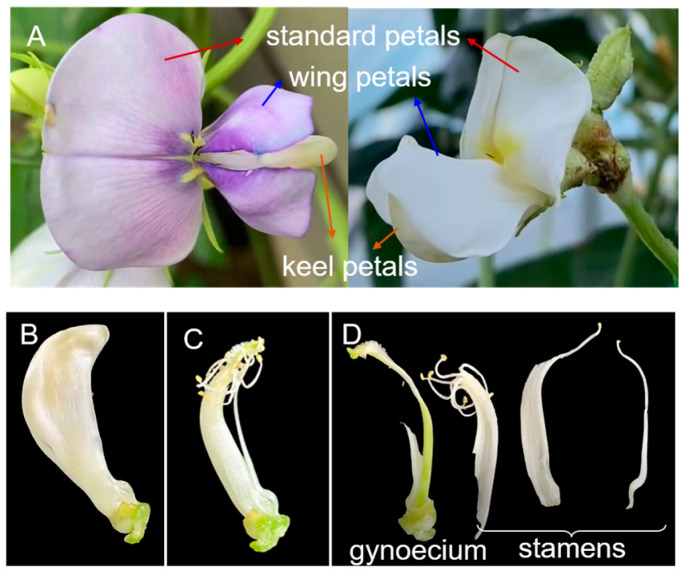
Structure of cowpea flowers (**A**): outside petals including standard petals (red arrows), wing petals (blue arrows) and keel petals (orange arrows); (**B**): keel petals; (**C**): the connate stamens (in a 9 + 1 arrangement) and gynoecium; (**D**): gynoecium and stamens.

Cowpea, *Vigna unguiculata* (L.) Walp (Fabaceae) is a nutritious crop used for human and livestock diets and is widely planted worldwide [31]. In China, thrips, including MTU, *Frankliniella intonsa* (Trybom 1895) and WFT [32], are among the major pests infesting cowpea, especially during the flowering period. The cowpea flower exhibits a zygomorphic papilionate structure, consisting of a large standard petal, two smaller lateral wing petals, and lower keel petals that enclose the inner connate stamens and gynoecium (Figure 1). Notably, keel petals remain enclosed during the whole blossom period, which may provide a shelter for thrips from exposure to pesticides, increasing the difficulty of thrips control [9]. However, the distribution of thrips within such structural flowers has yet to be clarified. Understanding plant structural traits and how thrips utilize them is critical for developing effective management strategies. Therefore, this study comprehensively investigated thrips distribution in structural flowers and assessed their abundance across floral developmental stages. The findings will contribute to understanding the spatiotemporal dynamics of thrips in relation to cowpea flower development, which is essential for improving thrips management strategies in cowpea production.

## 2. Results

### 2.1. Thrips Distribution Within a Cowpea Flower

**Figure 2:**
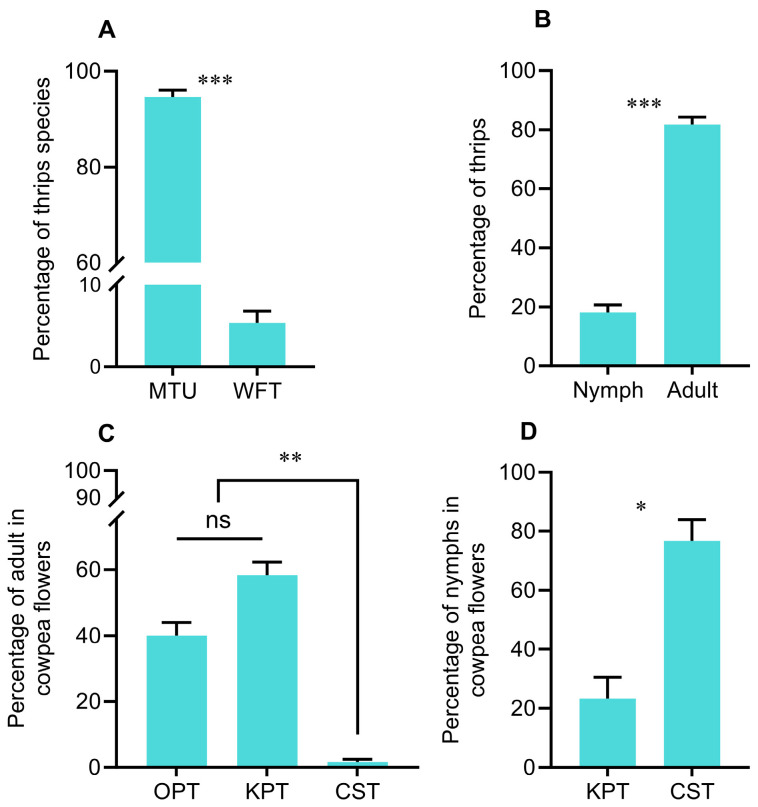
Adult species founded in cowpea flowers (**A**) (MTU and WFT), abundance of adult and nymphs (**B**), and distribution according to the floral architecture (**C**,**D**) indicated adult and nymph distribution in flowers (OPT—outside petals including the standard and wing petals, KPT—keel petals, and CST—inside of the connate stamens). Asterisks indicate significant differences (* *p* < 0.05, ** *p* < 0.01, *** *p* < 0.001), and ns indicates no significant difference (*p* > 0.05).

Adult MTU and WFT were identified in cowpea flowers, and the proportions were 94.62% and 5.38%, respectively (*t* = 24.117, *df* = 9, *p* < 0.001) (Figure 2A). The number of adults was significantly higher than that of nymphs (*t* = 7.598, *df* = 9, *p* < 0.001) (Figure 2B). Adults were mainly distributed in the keel petals (KPT), accounting for 58.28%, and 40.03% were distributed in the outside petals (OPT: standard petals and wing petals); only 1.69% of adults were found in the connate stamens (CST) (*χ*^2^ = 12.797, *df* = 2, *p* = 0.002) (Figure 2C). However, 76.68% of nymphs were in the CST, and only 23.32% were found in the KPT (*t* = −3.382, *df* = 5, *p* = 0.020) (Figure 2D).

### 2.2. Abundance of Thrips in Different Flower Ages

**Figure 3:**
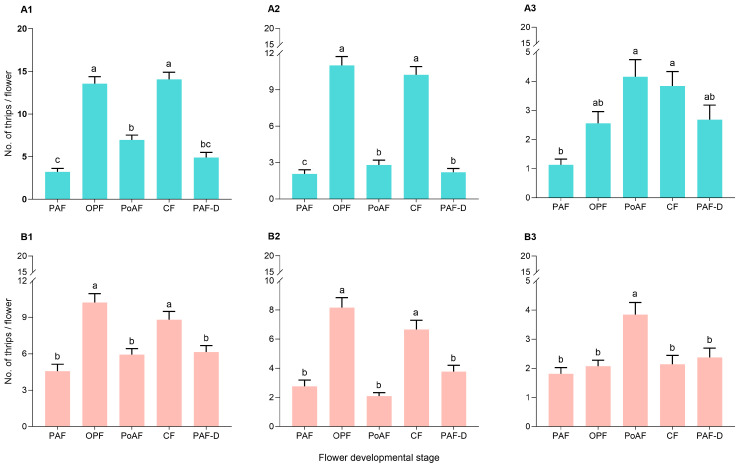
Abundance of total (**A1**,**B1**), adult (**A2**,**B2**), and nymphal (**A3**,**B3**) thrips in different floral development stages (PAF: preanthesis flowers in the morning; OPF: opening flowers; PoAF: postanthesis flowers; CF: closed flowers at dusk; PAF-D: preanthesis flowers at dusk) in 2023 (**A1**–**A3**) and 2024 (**B1**–**B3**). Different lowercase letters indicate statistically significant differences (*p* < 0.05).

The results show that flower stage affected thrips abundance (Figure 3). No significant difference was observed in the numbers of total and adult thrips in OPF in the morning and CF at dusk both in 2023 and 2024, exhibiting a significant difference to than that of PAF, PoAF in the morning, and PAF-D at dusk (Figure 3(A1): *F*_4,45_ = 39.96, *p* < 0.001; Figure 3(A2): *F*_4,45_ = 59.66, *p* < 0.001; Figure 3(B1): *F*_4,45_ = 6.570, *p* < 0.001; Figure 3(B2): *F*_4,45_ = 4.673, *p* = 0.003). Although no significant difference was observed among nymphal density on PoAF, CF, PAF-D, and OPF, the density of nymphs was the highest on PoAF in 2023, and significantly higher than that on PAF (Figure 3(A3): *F*_4,45_ = 4.766, *p* = 0.003). In 2024, the number of nymphs on PoAF was the highest, and no significant difference was observed among the number of nymphs on PAF, OPF, CF, and PAF-D (Figure 3(B3): *F*_4,45_ = 9.643, *p* < 0.001).

### 2.3. Behavior of MTU to Flowers

**Figure 4:**
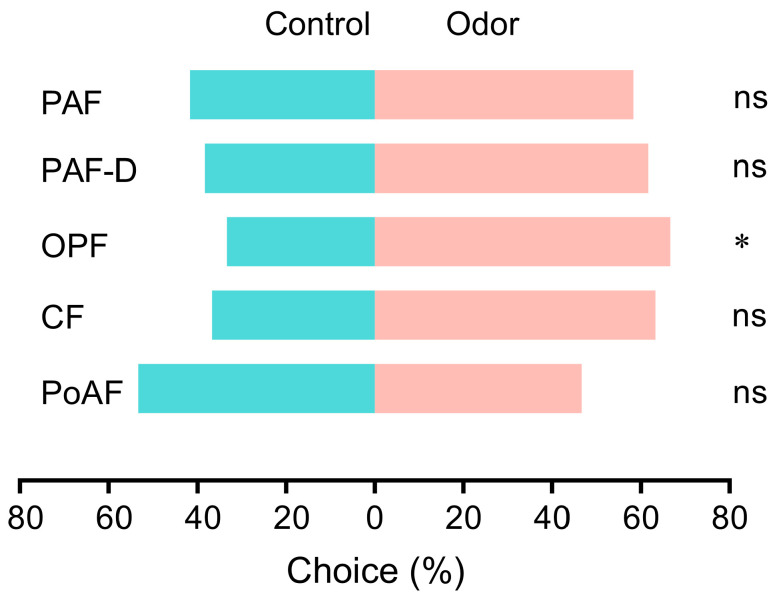
Behavioral responses of MTU to odors emitted by different flower stages (PAF: preanthesis flowers in the morning; OPF: opening flowers; PoAF: postanthesis flowers; CF: closed flowers at dusk; PAF-D: preanthesis flowers at dusk). An asterisk indicates a significant difference between odor sources (* *p* < 0.05), and ns indicates no significant difference between odor sources (*p* > 0.05).

The behavioral responses of female MTU to odors emitted by different flower stages were studied in a Y-tube olfactometer. The results show that MTU were significantly attracted by OPF odor (*χ*^2^ = 6.02, *df* = 1, *p* < 0.014) compared to the control (Figure 4). There was no statistically significant preference of MTU to odors of PAF, PAF-D, CF, and PoAF, while 61.7% and 63.3% MTU chose PAF-D and CF, respectively.

### 2.4. Differential Chemical Compounds in Flowers

The chemical compounds of floral volatiles emitted in different development stages were analyzed by GC-MS. A total of 11 compounds were identified (Table 1). Neophytadiene was greatly emitted from OPF. (*E*)-3-decenoic acid emitted from flowers in PAF and PAF-D, and 6,10,14-trimethyl-2-pentadecanone was specific in PoAF flowers. From PAF-D, OPF, to CF, 3-methyl-undecane was identified, and was greatly emitted from OPF and CF.

**Figure 5:**
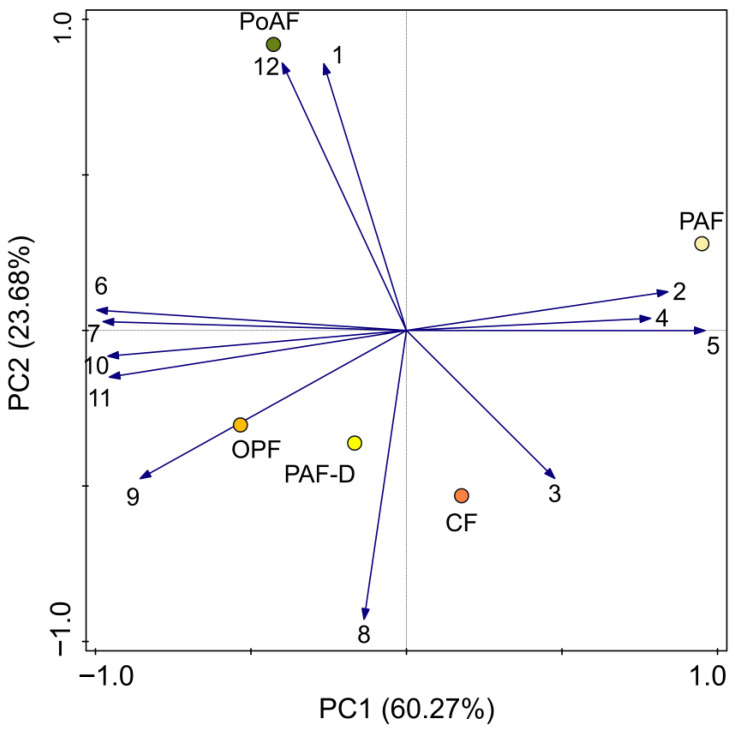
Principal component analysis (PCA) of flower volatiles during different stages. The numbers are the identified chemicals in Table 1. Squares represent the samples from different floral stages (PAF: open the following morning; PAF-D: preanthesis flowers open in the following morning; OPF: open flowers; CF: closed flowers, opened in the morning and closed at afternoon; PoAF: postanthesis flowers, open the previous morning).

Based on the principal component analysis (PCA) biplot, the volatile profiles of cowpea flowers exhibited clear stage-dependent clustering along the first two principal components, which together explained 83.95% of the total variance (PC1: 60.27%; PC2: 23.68%) (Figure 5). (*E*)-3-decenoic acid (code 2, shown in Table 1) and hexadecane (code 4), which showed high positive loadings on PC1, were strongly associated with pre-anthesis flowers (PAF). In contrast, ethyl (*E*)-4-decenoate (code 1) and hexahydrofarnesyl acetone (code 12), with high positive loadings on PC2, were closely linked to post-anthesis flowers (PoAF). Open flowers (OPF) and closed flowers (CF) formed a tight cluster, reflecting high similarity in their volatile composition. OPF were primarily characterized by ethyl (*Z*)-4-decenoate (code 9), while CF were associated with 3-methyl-undecane (code 3).

### 2.5. Speculation on Adult Thrips Dynamics in Cowpea Flowers

**Figure 6:**
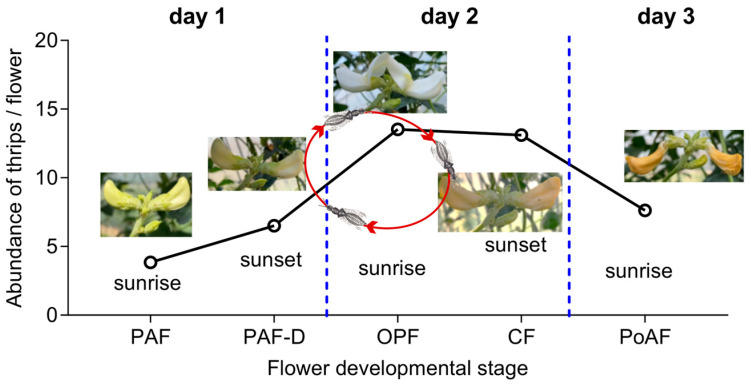
Simulation of switching of thrips in different flower developmental stages.

By integrating field surveys on thrips distribution across floral developmental stages with Y-tube olfactometer assays, we inferred that adult thrips exhibit a host-switching behavior associated with cowpea flower development (Figure 6). Adult thrips altered their host selection in accordance with floral development: thrips abundance in floral buds was low on the first day, but increased significantly when flowers opened the following morning. In the afternoon of the second day, thrips were found to be predominantly concealed with closed flowers. By the next morning (day 3), thrips, particularly adults, had abandoned senescing flowers and migrated to newly opened blossoms.

## 3. Discussion

### 3.1. Spatial Distribution Within a Cowpea Flower

Plant morphological traits vary across species as evolutionary adaptations to environmental conditions and can be developed by insects for feeding or development [33]. In the present study, the results show that thrips can fully utilize the architectural traits of cowpea flowers for survival and reproduction. Due to their cryptic feeding trait and small body size [6], thrips tend to hide in keel petals (Figure 2C). Furthermore, nymphs aggregate around the connate stamens and gynoecium (Figure 2D), where they can feed on nutrient-rich nectar and pollen [17,34], thereby facilitating thrips development. The arrangement of stamens in cowpea is noteworthy: nine stamens are fused together, while the tenth remains separate but adjacent, creating a distinct gap between the two groups (Figure 1C,D). This structural gap may coincidentally facilitate thrips penetration into the stamens. Additionally, other crevices, such as those at the junctions between sepals and petals, provide entry points for thrips to access keel petals or stamens, enabling them to reside within floral tissues [9]. Thus, the connate keel petals and fused stamens serve as shelters for thrips, and these refuge structures in cowpea flowers could hinder the efficacy of pesticides and biocontrol agents against thrips.

### 3.2. Volatile-Mediated Regulation of Thrips Abundance in Opening Flowers

Thrips were present in preanthesis, opening, and postanthesis flowers, but their abundance was highest in opening flowers. This pattern may be attributed to the variation in the floral scents [14,35], flower colors, shapes, and other visual traits [10,13,21,25]. Behavioral assays further indicated that MTU exhibited a strong preference for open flowers. Although MTU did not show a significant preference for postanthesis or closed flowers, more than 50% of individuals still selected these two flower types.

Previous studies show that volatile compounds from cowpea flowers are mostly aromatic [36], with several chemicals known to attract thrips [37]. For example, methyl anthranilate, a nitrogen-containing compound identified in cowpea flowers [36], has been demonstrated to be attractive to WFT, *T. tabaci* [38], *Thrips hawaiiensis* (Morgan 1913), and *Thrips coloratus* (Schmutz 1913) [39]. In the present study, however, only 12 volatile chemicals were identified across 5 different cowpea floral stages, which differs from findings reported in earlier research [36,40]. This discrepancy may largely be attributed to differences in collection methods. We immersed isolated flowers in hexane, which halts the continuous emission of volatiles from floral tissues, notably distinct from dynamic headspace extraction techniques for volatile collection [41]. Nevertheless, a relatively more volatile emissions were identified from the PAF-D, OPF, and CF stages than those at PAF and PoAF. Notably, heophytadiene was obviously emitted from OPF. Thus, these volatiles may assist thrips in locating opening flower, suggesting that the volatile profile of open flowers and their role in mediating thrips behavior warrant further investigation.

### 3.3. Potential Role of Color in Mediating Thrips Aggregation in Open Flowers

Cowpea flower colors can be classified into three main categories, purple/violet, yellow, and white [26,42,43], all of which have been reported to attract thrips [32,44]. The standard petals typically display a consistent bicoloration from base to tip [26], and this contrasting color pattern may function as a visual guide to attract insects [25,45,46,47]. Such colorful flowers often exhibit strong contrast against the surrounding environmental background, which consists largely of green leaves and stems. In the present study, cultivated cowpea flowers appeared predominantly white, yet yellow pigmentation was observed at the base of the standard petal and on the abaxial side of the wing petals (Figure 1 and Figure 6). Although the direct influence of color on thrips behavior was not quantified here, the intra-floral white-yellow contrast, together with the visual distinction between floral coloration and the green foliage of cowpea plants, likely enhances the attractiveness of these flowers to thrips.

### 3.4. Potential Movement Patterns of Adult Thrips in Cowpea Flowers

The exploitation of nutritionally diverse diets through host switching contributes to successful survival and reproduction in insects [13,14]. Cowpea flowers open in the morning and close around midday [26,42]. Although thrips numbers did not differ significantly between morning and dusk in preanthesis flowers, a sharp increase was observed in open flowers the following day, indicating that thrips relocate to open flowers between sunset and the next morning. Thrips abundance was highest in open flowers, and they were also observed sheltering in closed flowers at dusk. However, their populations declined substantially in postanthesis flowers the following day, suggesting a ‘leave-and-switch’ host-locating behavior in thrips from sunset to sunrise. Since thrips are not known to fly at night [34], we infer that adult thrips likely walk out of closed flowers and move to newly opened flowers in the early morning. Further surveys, including early morning across preanthesis, open, and postanthesis flowers, are needed to determine the exact timing of this flower switching behavior.

## 4. Materials and Methods

### 4.1. Field and Plants

Field studies were carried out at the Langfang Experiment Station of the Chinese Academy of Agricultural Science in Hebei Province during 2023 and at Linyi University in Shandong Province in 2024. In early June of both years, cowpea seeds (purchased from Shandong Shouhe Seed Industry Co., Ltd., Shouguang, China) were sown in beds, with each bed containing two rows. Within the same bed, the spacing between cowpea was 20 cm × 60 cm. Adjacent beds were separated by 50 cm, with each row measuring 20 m in length.

### 4.2. Thrips Species and Distribution Within the Opening Flowers

An investigation of thrips species and distribution within open flowers was conducted in mid-July 2023 during the flowering-to-pod developmental stage. Cowpea flowers open in the morning and closed in the afternoon; sampling was therefore carried out between 8:00 and 10:00 a.m., when flowers were in full bloom. Individual flowers were clipped while enclosed in sulfuric acid paper and immediately frozen at −20 °C for 15 min prior to thrips identification. Adult thrips from each flower were identified in the laboratory using an optical microscope (Olympus BX41, Olympus Corporation, Tokyo, Japan) with *Megalurothrips usitatus* (Bagrall 1913) (MTU) and *Frankliniella occidentalis* (Pergande 1895) (western flower thrips, WFT) being distinguished based on established morphological keys [16,48,49]. Each row was treated as one replicate. In total, 5–6 flowers were collected per replicate, with a total of 10 replicates. Similarly, 5–8 flowers were sampled per replicate, and 10 replicates were conducted to quantify the number of adults and nymphs per flower.

The distribution of thrips within the different floral structures—namely outside petals (OPT: standard and wing petals), keel petals (KPT), and the interior of connate stamens (CST)—was also investigated. In total, 6 replicates were carried out, with 6 flowers sampled per replicate.

### 4.3. Abundance of Thrips Within Floral Development Stage

In the morning, thrips abundance across three floral stages—preanthesis flowers (PAF, which open the following morning), open flowers (OPF), postanthesis flowers (PoAF, which opened the previous morning)—were investigated. At dusk (6:00–7:30 p.m.), counts were also performed on closed flowers (CF: flowers that opened in the morning and closed in the afternoon) and preanthesis flowers (PAF-D: which open the next morning). For each floral state, 10 replicates were performed, with 5–8 flowers sampled per replicate.

### 4.4. Thrips Behavior Assays to Flower Volatiles

According to the results of the investigation, the responses of female adult MTU to odors emitted by different floral states were assessed using the Y-tube olfactometer. The glass Y-tube consisted of two 5 cm long arms and a 5 cm long base tube, each with an inner diameter of 0.5 cm [50]. Filtered air was supplied at a flow rate of 300 mL/min per arm, passing through a charcoal filter before entering the odor source and then directed into the arms. Odor sources were prepared using three flowers at the same developmental stage. A single 2–3-day-old female adult after emergence starved for 4 h was introduced into the base tube of Y-tube using a fine brush. The responses to odor sources was recorded over a 3 min observation period; individuals that did not move up to 1/3 of the arm length within this time was excluded. To minimize positional bias, the orientation of the arms was reversed after every five runs, and both the Y-tube and odor sources were replaced after testing 10 individuals. Seventy-five individuals were tested for each odor pair. The control treatment consisted of no floral odor (CK), and the choices of MTU between CK and each floral odor (CK vs. PAF, CK vs. OPF, CK vs. PAF-D, CK vs. CF, and CK vs. PoAF) were assessed. Before trails, the Y-tube, odor bottles, and connecting tubes were rinsed with ethanol (Sinopharm Chemical Reagent Co., Ltd., Shanghai, China), followed by ultrasonic cleaning for 20 min. All glass components were then baked in a laboratory oven at 180 °C for 4 h to eliminate residual volatile contaminants. All bioassays were conducted between 08:00 and 18:00.

### 4.5. Collection of Flower Volatiles

Floral volatiles during bloom were collected via hexane immersion. Cowpea plants were grown in pots within a controlled-environment chamber under the following conditions: temperature 28 °C, relative humidity 60%, and a 16:8 h light–dark photoperiod. For each replicate, three flowers at the same developmental stage were immersed in 5 mL of hexane (Shanghai Macklin Biochemical Co., Ltd., Shanghai, China) for 4 h. After flower removal, the solution was concentrated to 300 µL under a gentle nitrogen stream, with 7.80 μg of ethyl caprate (Shanghai Macklin Biochemical Co., Ltd.) added as the internal standard. The extracts were stored at −20 °C. This collection procedure was repeated 4 times for flowers at different blooming stages (including PAF, PAF-D, OPF, CF, and PoAF).

### 4.6. Analysis of Extracts

The extracts were analyzed using a gas chromatograph-mass spectrometer (GCMS-QP2010 SE, Shimadzu, Tokyo, Japan) equipped with an HP-5 MS capillary column (30 m × 0.25 mm i.d. × 0.25 μm, Agilent Technologies Inc., Santa Clara, CA, USA). The GC-MS procedure was modified based on the method described by Osei-Owusu et al. (2020) [40]. The injector and transfer line temperature were set to 250 °C, and the ion source temperature was 230 °C. The MS scan range was 50–650 atomic mass units. After injecting 1 µL of the extract, the oven temperature was held at 60 °C for 1 min, ramped up to 230 °C at 6 °C/min, and then maintained for 5 min. Volatile compounds were tentatively identified by comparing with NIST 2014 mass spectral library. Co-injection with commercial compounds was performed for further verification.

### 4.7. Data Analysis

Adult MTU and WFT were identified in cowpea flowers. A paired *t*-test was used to compare the proportion of the two adult species, as well as the proportion of adults versus nymphs within an open flower. The Kruskal–Wallis test was used to compare the proportion of adult thrips among different floral structures (OPT, KPT, and CST). Because only 3 nymphs were found on OPT across all sampled flowers, nymph distribution on this structure was omitted; a paired *t*-test was therefore applied to compare nymph distributions between KPT and CST. The number of thrips—total and adults separately—in flowers at different developmental stages (PAF, PAF-D, OPF, CF, and PoAF) were compared using one-way analysis of variance (ANOVA) with Tukey’s HSD post hoc test. Data from the Y-tube olfactometer bioassay were analyzed with the chi-square test. Chemical compounds from different flower stages were compared with one-way ANOVA (with Tukey’s HSD post hoc test). Principal component analysis (PCA) was performed on mean-centered data to determine whether different floral developmental stages could be discriminated based on their volatile chemical profiles. All statistical analyses were performed using SPSS 21.0 (SPSS Inc., Chicago, IL, USA) at a confidence level of 0.05.

## 5. Conclusions

This study presents a comprehensive and detailed investigation of thrips distribution within cowpea flowers throughout their blooming stages. Thrips conceal themselves within cowpea flowers and move among flowers of different stages. Their preference for hiding in keel petals likely shields them from contact with sprayed pesticides or entomopathogenic fungi. Therefore, strategies such as ‘lure-and-kill’ or ‘lure-and infect’ [51,52] could be explored to reduce thrips abundance in cowpea flowers.

## Figures and Tables

**Table 1:** Volatile components from different floral development stages *.

Code	Component	PAF	PAF-D	OPF	CF	PoAF
1	ethyl (*E*)-4-decenoate	0.369 ± 0.045 a	0.371 ± 0.222 a	0.630 ± 0.250 a	0.380 ± 0.228 a	0.454 ± 0.180 a
2	(*E*)-3-decenoic acid	0.148 ± 0.037 a	0.032 ± 0.019 b	-	-	-
3	3-methyl-undecane	-	0.077 ± 0.0157 b	0.106 ± 0.042 a	0.209 ± 0.126 a	-
4	hexadecane	0.286 ± 0.037 a	0.260 ± 0.037 a	0.165 ± 0.067 a	0.442 ± 0.150 a	0.176 ± 0.034 a
5	octadecane	0.365 ± 0.044 a	0.297 ± 0.037 a	0.233 ± 0.095 a	0.399 ± 0.070 a	0.173 ± 0.029 a
6	neophytadiene	0.110 ± 0.003 c	0.248 ± 0.061 b	0.359 ± 0.03 a	0.249 ± 0.159 b	0.191 ± 0.029 bc
7	3-methyl-dodecane	0.076 ± 0.045 a	0.193 ± 0.064 a	0.249 ± 0.098 a	0.187 ± 0.112 a	0.158 ± 0.066 a
8	n-hexadecanoic acid	0.046 ± 0.027 a	0.195 ± 0.117 a	0.165 ± 0.022 a	0.193 ± 0.059 a	0.026 ± 0.026 a
9	ethyl (*Z*)-4-decenoate	-	0.082 ± 0.049 a	0.226 ± 0.090 a	0.119 ± 0.071 a	0.043 ± 0.017 a
10	(*E*)-2-dodecenol	-	0.041 ± 0.005 a	0.075 ± 0.032 a	0.013 ± 0.008 a	0.026 ± 0.026 a
11	2-nonen-1-ol	-	0.126 ± 0.025 a	0.158 ± 0.061 a	0.087 ± 0.017 a	0.093 ± 0.023 a
12	hexahydrofarnesyl acetone	-	-	-	-	0.047 ± 0.028

* Proportions (%) of peak areas to internal standard compound (ethyl caprate, 8.63 µg/300 µL hexane). Volatiles extracted from cowpea preanthesis flowers (PAF, open the following morning), preanthesis flowers (PAF-D, open in the following morning), open flowers (OPF), closed flowers (CF, opened in the morning and closed at afternoon), and postanthesis flowers (PoAF, open the previous morning). A dash indicates the compound was not found. Different lowercase letters within the same row indicate statistically significant differences. (*p* < 0.05).

## Data Availability

The data presented in this study are available on request from the corresponding author.
